# The epidemiology and etiology of adhesive capsulitis in the U.S. Medicare population

**DOI:** 10.1186/s12891-021-04704-9

**Published:** 2021-09-27

**Authors:** Sara M. Sarasua, Sarah Floyd, William C. Bridges, Stephan G. Pill

**Affiliations:** 1 School of Nursing, Clemson University, 436 Edwards Hall, Clemson, SC USA; 2 Department of Public Health Sciences, Clemson University, Clemson, SC USA; 3 Center for Effectiveness Research in Orthopaedics, University of South Caro, Greenville, SC USA; 4 School of Mathematical and Statistical Sciences, Clemson University, Clemson, SC USA; 5 Steadman Hawkins Clinic of the Carolinas, Prisma Health, Greenville, SC USA

**Keywords:** Adhesive capsulitis, Frozen shoulder, Stiff shoulder, Risk factors, Epidemiology

## Abstract

**Background:**

Adhesive capsulitis (AC) of the shoulder, also known as frozen shoulder, causes substantial pain and disability. In cases of secondary AC, the inflammation and fibrosis of the synovial joint can be triggered by trauma or surgery to the joint followed by extended immobility. However, for primary AC the inciting trigger is unknown. The burden of the disorder among the elderly is also unknown leading to this age group being left out of therapeutic research studies, potentially receiving delayed diagnoses, and unknown financial costs to the Medicare system. The purpose of this analysis was to describe the epidemiology of AC in individuals over the age of 65, an age group little studied for this disorder. The second purpose was to investigate whether specific medications, co-morbidities, infections, and traumas are risk factors or triggers for primary AC in this population.

**Methods:**

We used Medicare claims data from 2010–2012 to investigate the prevalence of AC and assess comorbid risk factors and seasonality. Selected medications, distal trauma, and classes of infections as potential inflammatory triggers for primary AC were investigated using a case–control study design with patients with rotator cuff tears as the comparison group. Medications were identified from National Drug codes and translated to World Health Organization ATC codes for analysis. Health conditions were identified using ICD9-CM codes.

**Results:**

We found a one-year prevalence rate of AC of approximately 0.35% among adults aged 65 years and older which translates to approximately 142,000 older adults in the United States having frozen shoulder syndrome. Diabetes and Parkinson’s disease were significantly associated with the diagnosis of AC in the elderly. Cases were somewhat more common from August through December, although a clear seasonal trend was not observed. Medications, traumas, and infections were similar for cases and controls.

**Conclusions:**

This investigation identified the burden of AC in the US elderly population and applied case–control methodology to identify triggers for its onset in this population. Efforts to reduce chronic health conditions such as diabetes may reduce seemingly unrelated conditions such as AC. The inciting trigger for this idiopathic condition remains elusive.

## Background

Adhesive capsulitis (AC), also known as frozen shoulder, is a common and debilitating shoulder condition, yet its cause remains largely unknown. The prevalence is typically reported to be 2–5% [1, 2], although estimates range as low as 0.5% [3] or as high as 10% [4]. The condition is most common among adults aged 40 to 70 years, but reported ages range from 27 to 85 [5]. Symptoms of this condition are shoulder joint pain with motion or pain during sleep followed by stiffness and dramatically reduced range of motion. The disability in the shoulder can last over a period of one or more years during which range of motion is slowly restored. For some patients, full range of motion is not restored even after four years [5, 6]. The typical time course for AC follows three stages [2]: a painful “freezing” stage where pain precedes reduced range of motion lasting 10–36 weeks. The next stage, lasting 4–12 months, is the “frozen” stiff stage where pain is reduced but range of motion remains impaired. The final stage, lasting 5–26 months, is the “thawing” stage where range of motion is gradually improves.

The pathophysiology of AC supports the theory that the condition results from a complex chain of events starting with inflammation leading to fibrosis and contracture of the shoulder capsule, the so-called “inflammatory-fibrotic cascade” [7]. During this cascade, inflammation, with cytokine proliferation lead to increased fibroblast proliferation, increased vascularization and new nerve growth, which may explain the pain reported by patients [7–12]. These factors in turn lead to increased and dysregulated collagen fiber deposition. Collagen fibers adhere to the glenohumeral ligaments, tendons, and joint surfaces causing contracture and joint stiffening. Thus, even after the inflammation is reduced, the adhesions remain greatly restricting motion [8, 9, 12]. It remains to be discovered what the inciting factor is that triggers this cascade and leads to the acute onset of AC.

AC is typically referred to as “primary” or “secondary”. Primary AC is idiopathic AC where examination and history cannot explain the development of the condition [2] whereas secondary AC develops due to pre-existing shoulder disorders or shoulder trauma [13] or post-mastectomy from breast cancer [14]. Primary AC is idiopathic with the cause unknown. Several risk factors for primary AC have been identified including diabetes [5, 15–17], thyroid disorders [15, 16, 18, 19], rheumatoid arthritis [13], gout [20], hyperlipidemia [17, 21] and Parkinson’s disease [22].

### Rationale

Despite what is known about the pathophysiology of AC and the associated risk factors, the trigger that sets primary frozen shoulder syndrome in motion is largely unknown and few studies have been undertaken to investigate triggers. There have been several small studies that suggest that the trigger for AC may be the usage of systemic medications, distal trauma, or perhaps there is a seasonal component that directly or indirectly initiates the disorder. Much of the sparse literature related to potentially medication-associated AC is based upon case reports or small case series that merit follow-up in larger samples [23–31]. Trauma has been suggested to generate systemic inflammation affecting areas distant from the site of trauma [32]. Another line of evidence supporting a possible role for infection as a trigger is the suggestion of seasonal occurrence of AC. The classic investigation by Nevasier (1945) reported 7 out of 8 patients had an onset during the winter months [33], suggesting a seasonal exposure akin to seasonality observed for influenza. Spouses are reported to have AC co-occur somewhat more than siblings [5] suggesting a shared environmental exposure as a possible risk factor. This observation by Hand et al. (2008) has received little notice or follow-up. Most recently, Ascani et al. (2021) report an increase in AC after COVID-19, supporting a potential association between viral infection and AC [34].

AC has generally been described as a condition affecting late middle-aged adults, and little has been written about the condition in the elderly population. Diabetes is the best studied risk factor for AC and the prevalence of diabetes is much higher in the ≥ 65 year age group (26.8%) than in the 18–44 years (4.2%) or 45–64 year age group (17.5%) making this group potentially more at risk [35]. With a large and growing population of older adults in the US, investigating the prevalence and triggers for this condition are warranted. Therefore, in this investigation, the prevalence of AC in the US Medicare population was determined. Then, using the cases we identified as primary AC and a control group without AC, we explore the hypothesis that AC may be triggered by an infection, trauma, or medications.

## Methods

### Data and sample

This study used complete Medicare Part B (fee-for-service) administrative claims data from the years 2010–2012 for all Medicare beneficiaries diagnosed with AC in 2011 (*N* = 92,437). The use of complete Medicare administrative data enabled patient healthcare utilization to be tracked across inpatient and outpatient settings and covered patients from all states in the United States. Participants in Medicare Part C, also known as Medicare Advantage, is a separately administered program and the data were not available for analysis. This project was reviewed by the University of South Carolina Institutional Review Board and determined to be not human research.

### AC prevalence cohort

Prevalence of AC (primary or secondary) in the Medicare population was determined by identifying Medicare beneficiaries with at least one diagnosis of AC in 2011 as defined by having an International Classification of Disease, Ninth Revision, Clinical Modification (ICD-9-CM) codes: 726.00, using Medicare Part B carrier claims. To calculate the denominator for prevalence estimates, the Centers for Medicare and Medicaid Services published data were used to determine the total population of Medicare beneficiaries over age 65 (40,685,908 people) and the subset of those with fee-for-service Part B coverage (26,756,183 people) [36]. Approximately two-thirds of Medicare beneficiaries over age 65 are covered by Medicare Part B.

### Primary AC cohort

As this study focuses on identifying triggers for new, primary AC diagnoses, a subset of the AC prevalence cohort representing those Medicare beneficiaries with primary AC was identified. In order to best identify those beneficiaries with primary AC, a series of clinically guided inclusion criteria were applied. First, all Medicare beneficiaries with an index (first) shoulder visit in 2011 and no previous shoulder diagnoses in the 365-days prior were identified. This included 192 ICD-9-CM shoulder related diagnosis codes, including AC and RCT. The index shoulder date was defined for each beneficiary as the first date where a shoulder diagnosis was received in 2011. Of that group, individual beneficiaries with a diagnosis of AC (ICD-9-CM codes: 726.00) within 90 days of the index shoulder visit and an x-ray or MRI within 30 days of the index shoulder visit were included. Given that diagnostic imaging is routinely used in the AC diagnosing process to rule out other clinically similar shoulder diagnoses, this inclusion requirement increased the confidence in the diagnosis of AC and the likelihood the case was receiving an initial diagnosis of AC. Following the diagnostic imaging service, beneficiaries also had to have a subsequent AC diagnosis claim within 180 days of their first AC diagnosis claim. The selection of the 30, 90, and 180 day windows was based upon expertise of the investigators and the typical pattern of care when making a new diagnosis.

**Fig. 1:**
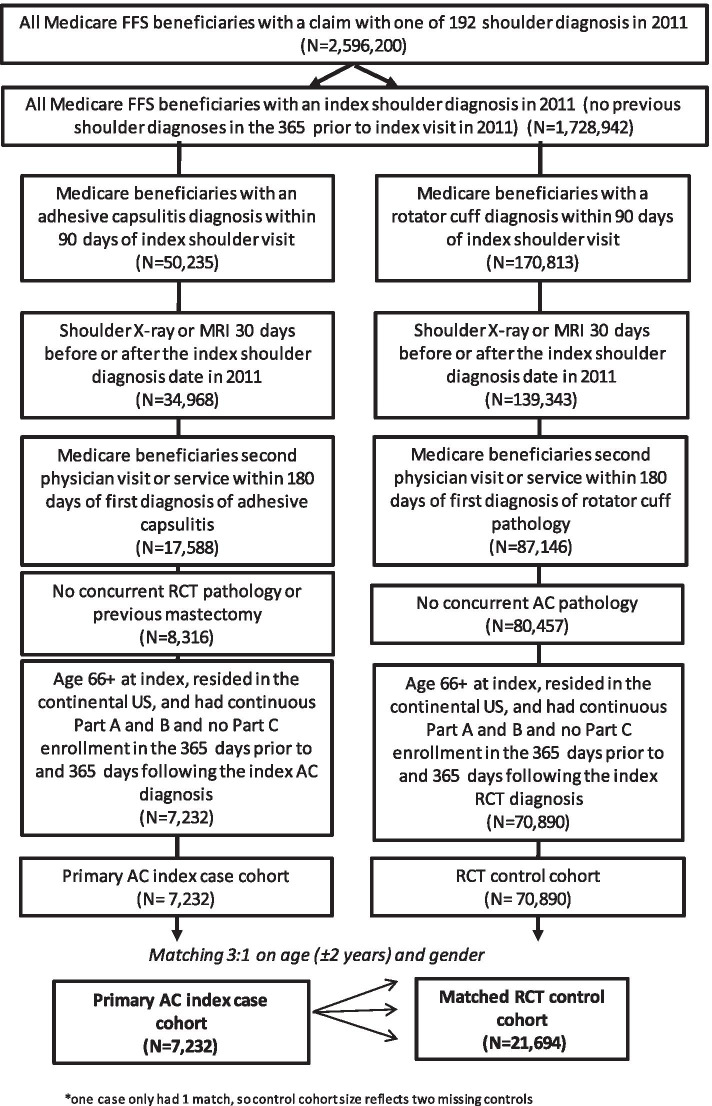
Flow chart of the selection of cases and controls. FFS- Fee-for-service beneficiaries. Does not include Medicare Advantage plan participants

Given the known direct association between mastectomy and AC diagnosis [14], any beneficiaries that had a mastectomy (CPT 85.2, 85.3, 85.4) were excluded from the analysis. Patients with fracture, dislocations, and sprains involving the shoulder were also excluded as these are known causes of secondary AC [13]. Furthermore, because rotator cuff (RC) tears were used as our control group, any AC beneficiaries who had a concurrent RC diagnosis were also excluded from the study. The presence of co-morbid chronic health conditions such as diabetes were not exclusion criteria as these factors are considered risk factors and not acute triggers. Additional inclusion criteria applied to assure complete data included (1) continuous enrollment in fee-for-service Medicare Part A and Part B from 365-days prior to 365-days after the index shoulder diagnosis and no enrollment in Medicare Part C (also known as Medicare Advantage plans) during the study period, (2) aged 66 years on the index shoulder visit, and (3) residence within the continental United States or Hawaii. The final residency criteria excludes Medicare beneficiaries who may obtain medical care outside the U.S. Medicare system and thus would have incomplete data. The minimum age criterion of 66 years was used to ensure enrollment in the Medicare system for a year prior to the index shoulder diagnosis. Complete patient inclusion criteria can be found in Fig. 1. The final primary AC case cohort included 7,232 Medicare beneficiaries.

### Rotator cuff controls selection

A group of beneficiaries with a rotator cuff tear diagnosis in 2011 were selected as controls in our study. Rotator cuff tear was selected because of its similar clinical presentation but distinct etiology, and because it has been used as a control group in two studies of AC [37, 38]. Similar inclusion criteria were applied to identify the control group using Medicare Part B claims. Individual patients with a diagnosis of rotator cuff tear (ICD-9-CM codes: 727.61, 840.3, 840.4 covering partial thickness tears, bursal-sided tears, full thickness tears, massive tears) within 90 days of the index shoulder visit and an x-ray or MRI within 30 days of the index shoulder visit were included. Medicare beneficiaries also had to have a subsequent rotator cuff tear diagnosis claim within 180 days of their first rotator cuff diagnosis claim within 2011. Patients with any shoulder diagnosis in the 365-days prior to their index shoulder diagnosis in 2011 were excluded from the study. Additionally, any beneficiaries that had a concurrent diagnosis of AC were excluded from the control cohort. Additional inclusion criteria applied to assure complete data included continuous enrollment, aged 66 years on the index shoulder data, and residence within the continental United States. The final rotator cuff tear control cohort included 70,890 eligible Medicare beneficiaries.

### Case–control matching process

Controls were matched to cases in a 3:1 ratio based on beneficiary age within 4 years (± 2 years) and gender. One older case was only matched to one control due to limitations in control sample at the age range. The final matched RC control group was 21,694 beneficiaries. Cases and controls were selected according to the flow chart described in Fig. 1.

### Medication and vaccine measures

Medication use in the 180 days prior to index shoulder diagnosis was obtained from Medicare National Drug Code (NDC) data and translated to the World Health Organization (WHO) Anatomical Therapeutic Chemical (ATC4) classification code [39] using the cross-reference coding developed by Kury et al. [40]. An exposure period of 180-days prior to index shoulder diagnosis was chosen to allow time for initiation of the inflammatory pathway as well as account for delays between onset of AC and accessing medical care. Prior case reports indicated wide variation in time between initiation of medication and onset of AC, such as 10 days for fluoroquinolones [26] and 3 to 5 months for primidone, metalloproteinase inhibitors, and acitretin [25, 27, 30]. Six drug classes previously noted in the literature to increase the risk of AC were studied: fluoroquinolones [25], barbiturates [24], antituberculosis agents [31], protease inhibitors [28, 29], retinoids [27], and metalloproteinase inhibitors [30]. An additional class of antiviral agents was examined to serve as a potential proxy measure for exposure to a viral agent.

The hierarchical nature of the ATC4 code allowed classification into categories of medication and specific usages. The following ATC4 codes were used: J01M to capture quinolones, J04A for antimycobacterials to treat tuberculosis including isoniazid, N03AA for any barbiturate, D05BB and D10BA for retinoids for systemic use, B02AB for proteinase inhibitors, J05AE for protease inhibitors, and J05A for antivirals. Medication utilization was identified using Part D claims. Receipt of an influenza or pneumococcal vaccine in the 180 days prior to index diagnosis was determined from Healthcare Common Procedure Coding System (HCPCS) codes in Part B claims: 90,630, 90,653, 90,654, 90,655, 90,656, 90,657, 90,658, 90,660, 90,661, 90,662, 90,672, 90,673, 90,674, 90,682, 90,685, 90,686, 90,687, 90,688, 90,689, 90,756, Q2035, Q2036, Q2037, and Q2038.

### Infectious diseases and trauma exposure

Diagnosis codes were examined for infectious disease and trauma diagnoses occurring 180-days prior to the index shoulder diagnosis. Categories of infectious diseases were created following prior classification systems [13, 41, 42]. The five most common infectious disease groups, occurring in at least 3% of the study population were examined in the analysis. The minimum of 3% was selected for pragmatic reasons to identify a common trigger. With an estimate of 3% exposure in cases, the study had 99.6% power to detect an odds ratio of 1.5 or higher. The infectious disease groups included mycoses (ICD-9-CM codes 110.0–118.0), upper respiratory tract infections (ICD-9-CM codes 460.X-466.X, 475.X), lower respiratory tract infections (ICD-9-CM codes 480.X-489.X, 490.X, 510.X, 513.X), infections of the kidney and urinary tract (ICD-9-CM codes 590.0–590.9, 599.0, 595–595.9, 597.X), and skin infections (ICD-9-CM codes 680.X-686.X, 695.5).

Trauma categories excluded trauma to the shoulder and were divided into fracture, dislocation, or sprains (ICD-9-CM codes 800.X-848.X) and other traumatic injuries (ICD-9-CM codes 850.X-854.X, 870.X-879.X, 880.X-887.X, 890.X-897.X, 900.X-904.X, 920.X-924.X, 925.X-929.X, 950.X-957.X, and 959.X) based upon classification used by Peterson et al. [32].

### Patient characteristics and comorbid conditions

Patient demographic characteristics were measured by cross referencing the 2011 Beneficiary Summary files from Medicare. Specific patient-level variables included age, sex, race, and dual-eligibility for Medicare and Medicaid status on the index shoulder diagnosis date. General patient health was measured using Part A and B Medicare spending in the year prior to the index shoulder date, the Charlson Comorbidity Index (CCI), and the Frailty Risk Index (FRI). CCI is a validated measure of burden of disease [43–45]. Comorbidities are weighted from 1 to 6 for mortality risk and disease severity and then summed to form the total CCI score [43–45]. The FRI score is a validated instrument for assessing frailty among older persons [46]. Comorbid conditions that have been previous associated with AC were identified using ICD-9-CM codes as follows: thyroid disorder (240–246), Type 1 and Type 2 diabetes (250.XX-250.93), hyperlipidemia (272.0–272.9), hypertension (401–405), rheumatoid arthritis (714.0), and gout (274).

### Statistical analysis

Descriptive statistics were calculated for determining the overall prevalence of AC and the demographics of the AC cohort. Then descriptive statistics, followed by t-tests or Chi-square tests, were used to assess the association of AC presence and the risk factors (infections, traumas, medications, and patient demographics) individually. A *P*-value < 0.05 was considered statistically significant. Seasonality of AC presence was assessed by estimating the proportions of new primary AC cases diagnosed per month with 95% confidence intervals, and using the intervals to identify months that were higher or lower than typical. Logistic regression models were used next to assess the association with AC and the risk factors in a combined model. Logistic regression results were presented as 95% confidence intervals for the odds ratios. A 95% confidence interval for odds ratios that excluded 1.0 was considered to be evidence of a statistically significant association. Because the case–control matching was performed on only age and gender (and thus considered to be a loose match), unconditional logistic regression was performed [47]. A conditional model was also run to verify similar results.

## Results

### Prevalence of AC in the US Medicare population

We found 92,437 individuals over age 65 with a diagnosis of AC in the Part-B beneficiary population, representing 4% of those with a shoulder claim. We estimate that the one-year prevalence for AC in the over 65-year age group to be 0.35% (92,437/26,756,183 Part-B beneficiaries). This prevalence of 0.35% translates to an estimated total 142,000 people over age 65 with AC across the U.S. (0.35% of the 40,685,908 total Medicare for the aged population). Average one-year Medicare spending, in the year prior to diagnosis, for those beneficiaries with a diagnosis of AC in 2011 was substantially higher ($14,176) than the average spending per Medicare beneficiary in 2011 ($10,756) [48].

Two thirds of AC patients were female, and the mean age of the AC cohort was 75.7 years (Table 1). White (86.3%), Black (6.7%), and Asian (2.8%) were the most common reported racial categories for patients with AC. Patients came from all regions of the United States, and two-thirds were younger than age 76 years. Using the Charlson Comorbidity Index as a measure of general health status, approximately 51.1% of individuals with AC had a CCI score of 2 or higher, indicating more co-morbidities [43, 45]. Common co-morbidities examined included diabetes (34.5%), hyperlipidemia (44.5%), hypertension (58.4%) and thyroid disorders (17.7%) while rheumatoid arthritis, gout, and Parkinson’s disease were present in less than 5% of patients (Table 1).

**Table.1:** Patient Characteristics for all 2011 U.S. Medicare Beneficiaries diagnosed with Adhesive Capsulitis

	All Adhesive Capsulitis Cases (*N* = 92,437)
	N (%)
*Male*	33,012 (35.7)
*Race*	
Asian	2,628 (2.8)
Black	6,199 (6.7)
Hispanic	1,620 (1.8)
Other	2,186 (2.4)
White	79,804 (86.3)
*Dual eligible for Medicaid*^*a*^	10,999 (11.9)
*Mean Age (Standard Deviation)*	75.7 (7.0)
*Age Group*	
66–69	24,026 (26.0)
70–75	29,143 (31.5)
76–79	14,380 (15.6)
80–85	15,628 (16.9)
86 +	9,260 (10.0)
*Region*	
Midwest	18,758 (20.3)
Northeast	19,310 (20.9)
South	37,440 (40.5)
West	16,334 (17.7)
*Charlson Comorbidity Index (CCI)*	
0	24,775 (26.8)
1	20,391 (22.1)
2	14,683 (15.9)
3	11,426 (12.4)
4 +	21,162 (22.9)
*Frailty Risk Index (FRI)*	
0	34,721 (37.6)
1	30,776 (33.3)
2	13,593 (14.7)
3 +	13,347 (14.4)
*Mean Previous Year Medicare Spending (SD)*^*b*^	$14,176 (23,287)
*Comorbidities*	
Diabetes, Type 1	3,220 (3.5)
Diabetes, Type 2	28,611 (31.0)
Hyperlipidemia	41,106 (44.5)
Hypertension	53,979 (58.4)
Thyroid Disorder	16,364 (17.7)
Rheumatoid arthritis	3,883 (4.2)
Gout	2,744 (3.0)
Parkinson’s Disease	1,299 (1.4)

^a^ Beneficiary was fully dual-eligible for Medicare and Medicaid during the month the index shoulder visit occurred

^b^ Total Part A and B payments made by Medicare for the beneficiary over the period of 365-days prior to their index shoulder date

### Case control study of incident cases of primary AC and rotator cuff tears as controls

The final sample for analysis included 7,232 individuals with newly diagnosed primary AC and 21,727 newly diagnosed rotator cuff tears matched to cases on age and gender. By design, gender and age distributions were the same with 35.8% male and a mean age of 74.8 years (Table 2). AC and rotator cuff patients came from all regions of the United States with a larger proportion of AC patients having a CCI score of 2 or more (45.3%) compared to only 41.2% of rotator cuff controls with a CCI score of two or more. Many factors reached statistical significance in comparing the two groups, but the actual differences in proportions between cases and controls were not clinically meaningful.

**Table.2:** Comparison of Patient Characteristics for Incident Adhesive Capsulitis Cases and Matched Rotator Cuff Tear Controls

	Adhesive Capsulitis Cases (*N* = 7,232)	Rotator Cuff Tear Controls (*N* = 21,694)	*p*
	N (%)	N (%)	
*Male*	2,592 (35.8)	7,776 (35.8)	0.99
*Race*			< 0.0001
Asian	133 (1.8)	175 (0.8)	
Black	453 (6.3)	1,037 (4.8)	
Hispanic	73 (1.0)	251 (1.2)	
Other	183 (2.5)	311 (1.4)	
White	6,390 (88.4)	19,920 (91.8)	
*Dual eligible for Medicaid*^*a*^	513 (7.1)	1,111 (5.1)	< 0.0001
*Mean Age (Standard Deviation)*	74.8 (6.8)	74.9 (6.7)	0.11
*Age Group, years*			0.04
66–69	2,278 (31.5)	6,449 (29.7)	
70–75	2,303 (31.8)	7,218 (33.3)	
76–79	1,011 (14.0)	3,073 (14.2)	
80–85	1,063 (14.7)	3,266 (15.1)	
86 +	577 (8.0)	1,688 (7.8)	
*Region*			< 0.0001
Midwest	1,641 (22.7)	4,909 (22.6)	
Northeast	1,449 (20.0)	4,019 (18.5)	
South	2,955 (40.9)	8,824 (40.7)	
West	1,136 (15.7)	3,849 (17.7)	
*Charlson Comorbidity Index (CCI)*			< 0.0001
0	2,369 (32.8)	7,754 (35.7)	
1	1,582 (21.9)	5,005 (23.1)	
2	1,045 (14.4)	3,444 (15.9)	
3	881 (12.2)	2,350 (10.8)	
4 +	1,355 (18.7)	3,141 (14.5)	
*Frailty Risk Index (FRI)*			< 0.0001
0	4,161 (57.5)	12,487 (57.6)	
1	1,822 (25.2)	5,699 (26.3)	
2	675 (9.3)	2,106 (9.7)	
3 +	574 (7.9)	1,402 (6.5)	
*Mean Previous Year Medicare Spending (SD)*^*b*^	$10,174 (20,995)	$8,130 (14,971)	

^a^Beneficiary was fully dual-eligible for Medicare and Medicaid during the month the index shoulder visit occurred

^b^Total Part A and B payments made by Medicare for the beneficiary over the period of 365-days prior to their index shoulder date

### Assessment of seasonality

**Fig. 2:**
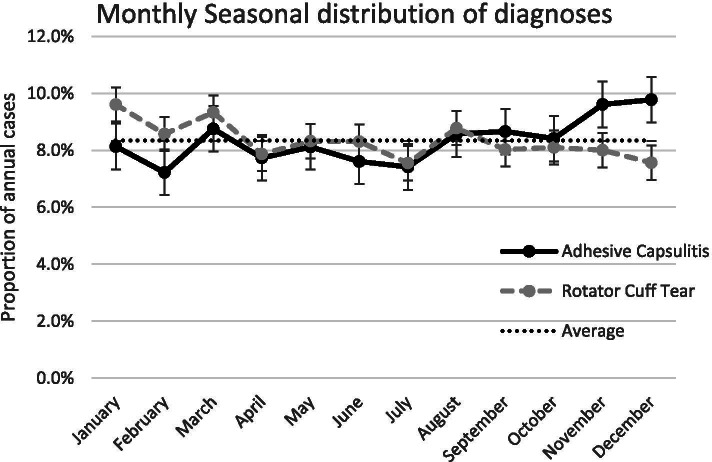
Monthly seasonal distribution of diagnoses for adhesive capsulitis and rotator cuff tears, adults ages 65 and older, 2011 U.S. Medicare Data. Error bars show 1 SD of 0.8% for AC and 0.6% for RCT. The average line shows the expectation of 8.3% of diagnoses to occur each month if there is no monthly variation

The proportion of new cases diagnosed by month was plotted for AC and rotator cuff tear patients (Fig. 2). While the highest proportion of AC cases were diagnosed in the last 5 months of the year, particularly in November and December (9.6% and 9.8%, respectively), these numbers did not exceed 2 standard deviations above the mean monthly proportion of 8.3% $\pm$ 1.6%. Similarly, there was no apparent seasonal trend for rotator cuff tears as all months were within the monthly mean of 8.3% ± 1.3%.

### Prevalence of comorbid health conditions

The prevalence of known co-morbid conditions and risk factors for AC were compared for primary AC cases and rotator cuff tear controls (Table 3). The odds of having AC was significantly higher for those beneficiaries with Type 1 diabetes compared to those without diabetes (odds ratios of 1.37 (95% CI 1.17–1.61) and for Type 2 diabetes (OR = 1.22 (95% CI 1.14–1.29). Parkinson’s disease, although uncommon, also showed a positive association with AC with an odds ratio of 1.58 (95% CI 1.23–2.04). Hypertension, hyperlipidemia, and thyroid disorders were prevalent for both cases and controls but no significant association was made with AC.

**Table.3:** Univariate and unconditional logistic regression analyses of the associations between medical comorbidities and diagnosis of adhesive capsulitis

	**Cases**	**Controls**	**Adjusted Odds Ratio**^**a**^	
*Medical Comorbidities*	N (%)	N (%)	OR	95% CI
Thyroid disorder	1,181 (16.3)	3,446 (15.9)	1.02	(0.95, 1.10)
Diabetes, Type 1	243 (3.4)	476 (2.2)	**1.37**	**(1.17, 1.61)**
Diabetes, Type 2	2,149 (29.7)	5,480 (25.3)	**1.22**	**(1.14, 1.29)**
Hyperlipidemia	3,242 (44.8)	9,543 (44.0)	1.03	(0.97, 1.08)
Hypertension	3,886 (53.7)	11,661 (53.8)	0.98	(0.93, 1.04)
Rheumatoid Arthritis	131 (1.8)	452 (2.1)	0.85	(0.70, 1.04)
Gout	181 (2.5)	471 (2.2)	1.13	(0.95, 1.34)
Parkinson’s Disease	92 (1.3)	175 (0.8)	**1.58**	**(1.23, 2.04)**

^a^Model was adjusted for age and gender

### Association with medications taken prior to diagnosis

The association with medications of a priori interest was investigated. After adjusting for age, gender, and the two significant comorbid conditions, diabetes and Parkinson’s disease, we found that odds of having AC was lower for individuals taking a quinolone antibiotic (OR = 0.88, 95% CI 0.79–0.97) in the 180-days prior to the index shoulder diagnosis (Table 4). No association was found for receiving an influenza or pneumococcal vaccination or taking a barbiturate or antiviral class of medication and having a diagnosis of AC. Recorded usage of barbiturates in the sample was low (0.3%). Too few individuals (< 10 per group) were identified who took retinoids, anti-mycobacterials, protease inhibitors, or metalloproteinase inhibitors to allow robust analysis in this investigation.

**Table.4:** Univariate and unconditional logistic regression analyses of the associations between medication utilization and diagnosis of adhesive capsulitis

	Cases	Controls	Adjusted Odds Ratio (OR)^a^	
*Medical Utilization*	N (%)	N (%)	OR	95% CI
Influenza or pneumococcal vaccine	2,301 (31.8)	6,788 (31.3)	1.02	(0.96, 1.08)
Quinolones	545 (7.5)	1,815 (8.4)	**0.88**	**(0.79, 0.97)**
Barbiturates	19 (0.3)	46 (0.2)	1.22	(0.71, 2.09)
Antivirals	106 (1.5)	382 (1.8)	0.85	(0.68, 1.06)

^a^Model was adjusted for age, gender, diabetes type 1, diabetes type 2 and Parkinson’s disease

### Association with infection or trauma prior to diagnosis

Five infectious disease classes occurred in at least 3% of the population in the 180 days prior to diagnosis and were examined as potential precipitating factors for AC (Table 5). Upper respiratory tract infections were common, occurring in 10.3% of people with AC and 12% in people with rotator cuff tears. People with AC were less likely to have had an upper respiratory infection prior to diagnosis than people with rotator cuff tears (OR = 0.84, 95% CI 0.77–0.92). Lower respiratory infections were less common at 3.5% of cases and controls. No association was observed for mycoses or infections of the lower respiratory tract, kidney or urinary tract, or skin prior to diagnosis. No significant difference was observed for having a non-shoulder related fracture or sprain or other trauma prior to diagnosis in the AC group compared to rotator cuff tear group. Approximately 6–7% of cases and controls experienced these types of trauma prior to diagnosis.

**Table.5:** Univariate and unconditional logistic regression analyses of the associations between infectious disease triggers, trauma and diagnosis of adhesive capsulitis

	Cases	Controls	Adjusted Odds Ratio (OR)^a^	
	N (%)	N (%)	OR	95% CI
*Infectious Triggers*				
Mycoses	703 (9.7)	1,895 (8.7)	1.09	(0.99, 1.20)
Upper respiratory	745 (10.3)	2,605 (12.0)	**0.84**	**(0.77, 0.92)**
Lower respiratory	256 (3.5)	761 (3.5)	1.05	(0.91, 1.22)
Kidney or urinary	640 (8.8)	1,782 (8.2)	1.09	(0.99, 1.20)
Skin infection	333 (4.6)	934 (4.3)	1.06	(0.93, 1.21)
*Trauma*^b^				
Fracture or sprain	442 (6.1)	1,451 (6.7)	0.91	(0.82, 1.02)
Other traumatic injuries	471 (6.5)	1,526 (7.0)	0.92	(0.83, 1.03)

^a^Model was adjusted for age, gender, diabetes type 1, diabetes type 2, Parkinson’s disease and quinolones

^b^Excludes shoulder-related traumas

## Discussion

### Background and rationale

While AC has been studied in middle-aged adults, less is known about its epidemiology in the elderly population. In addition, little is known about the inciting trigger that leads to the development of primary AC. Therefore, this case control study was undertaken to describe AC among the elderly and explore the hypotheses that exposure to selected medications, distal traumas, or seasonal factors, may increase the risk for AC.

#### Limitations

There were some limitations to this analysis. While three years of complete data were available, we were not able to assess trends beyond the one-year window into the year 2011. The use of claims data reflects dates of care rather than medical history. Date of claims diagnosis was used to identify date of onset, yet there may have been delay between symptom onset and seeking care. Information such as body mass index or obesity, detailed information on medication prescriptions, occurrence of infections not attended to by medical professionals, and confirmation of diagnosis of cases or controls may be less accurate using claims data than using medical records. The 7,232 new cases that were examined in detail may be an underestimate of the true number of new cases per year of primary AC, as our selection criteria required diagnostic imaging whereas not all healthcare providers order imaging to make the diagnosis. Further, patients were removed if they had any other shoulder diagnosis in the prior year which may have eliminated cases that had unrelated conditions and some cases may have been misdiagnosed with another disorder. However, the selection criteria were used in order to assess potential triggers for new cases which required a stringent selection criteria. One challenge in this analysis was selecting the most appropriate window to identify potential triggers. We selected a 6-month window prior to AC diagnosis because studies of selected medication-associated AC indicated most [25–27, 30], but not all [29], exposures occurred within six months of diagnosis. However, associations with long term or chronic use of medications may have been missed. Further, the window may have been too wide for association with infectious triggers given that reactive arthritis typically occurs within six weeks of infection [49]. Finally, we used patients with rotator cuff tears as a comparison group due to the similarities in presentation yet distinct etiology. Other investigations of AC have also used rotator cuff tears as comparators [37, 38]. However, the two disorders may share some underlying risk factors. In particular, fluoroquinolone [50, 51] and influenza vaccinations [52, 53] have been found to be associated with tendinopathy and rotator cuff tears. Future investigations may consider the use of additional comparison groups to investigate triggers for AC.

A strength of the study was that the prevalence of AC in the elderly population has not been well-studied to date. We included a large sample size representing all cases for an entire year across the United States among Medicare beneficiaries. The nearly universal use of Medicare among the elderly makes the findings representative of older American adults. The large sample size allowed the assessment of many potential risk factors, and the claims data included information on medications, concomitant diagnoses, and procedures.

#### Prevalence of AC in adults over 65 years of age

The first objective was to describe the epidemiology of AC in older adults, an age-group infrequently studied. Although AC is not typically thought to be a condition common among elderly adults, we estimate more than 140,000 Medicare beneficiaries were diagnosed with primary or secondary AC in 2011, accounting for 4% of shoulder diagnoses in this age group. We found women were more likely to have AC than men by a ratio of 1.8 to 1. Older age did not attenuate the risk for AC in women. Most people with AC in the study were white (86.3%), reflecting the demographics of the U.S. Medicare population. The patients with AC commonly had other co-morbid conditions including diabetes, hyperlipidemia, hypertension, and thyroid disorders. Rheumatoid arthritis, gout, and Parkinson’s disease were present in less than 5% of patients. These findings agree with findings from studies of younger populations [5, 15, 16], suggesting that the effects of age do not diminish the increased risk for women or those with chronic disease.

We also found that on average, Medicare annual spending for patients with AC was significantly higher than the average annual beneficiary Medicare spending in 2011 [48]. The growing elderly population and prevalence of AC in this age group suggests that AC can be a significant cause of disability for this population. Furthermore, the higher spending for beneficiaries diagnosed with AC might indicate that those beneficiaries are in worse health and require more healthcare utilization in addition to costly AC treatment.

#### Risk Factors for AC in adults over 65 years of age

The second objective was to investigate risk factors and potential triggers for AC. We examined newly diagnosed cases of AC and compared them to people with newly diagnosed rotator cuff tears. Age and gender were similar by study design. Elderly beneficiaries in the U.S. Medicare population are overwhelmingly white. However, a larger proportion of AC patients were non-white (11.6%) compared to those with rotator cuff tears (8.2%). This contrasts with earlier investigations which suggested a higher risk of AC for white patients [54] but is supported by more recent and larger studies which also found higher rates in non-white patients [55]. Others have observed higher rates for Asian ancestry individuals in particular [13, 56].

We found that both type 1 and type 2 diabetes as well as Parkinson’s disease were more likely in older patients with AC. Our findings corroborate that diabetes, a well-known risk factor for AC in the middle age population [13, 15–17, 55], is also associated with AC in the elderly population. The reason diabetes increases risk for AC is unclear, although possible biological mechanisms have been proposed. Hwang et al. identified increases in advanced glycation end products along with increased collagen density and fibroblast proliferation in capsular tissue of patients with AC and diabetes [38]. Their findings support the hypothesis that increased blood glucose levels increase the glycation of collagen proteins which then increases cross-linking of these fibers and stiffening of the joint. The association with Parkinson’s disease is less well established but confirms prior reports [22]. Contrary to initial expectations for increased risk, no association was observed for thyroid disorders.

#### Medication Utilization, infection and trauma as potential triggers

In addition to examining AC in the under-studied elderly population, the novel contribution of our work was to explore the association between plausible triggers (systemic medications, infections, seasonality, and inciting traumatic injury) and AC. One potentially treatment-related risk factor (use of quinolone antimicrobial treatment) was found to be significantly less common in patients with AC than rotator cuff tear controls. It is possible that fluoroquinolone exposure is associated with tendinopathy and possibly could contribute to rotator cuff tears [50]. The stronger association with rotator cuff tears prevents a true assessment of the risk of these factors on AC. A review of Medicare prescription drug usage shows approximately 20% of beneficiaries had a fluoroquinolone Part D claim in 2018 making it a potentially common risk factor. Receipt of an influenza or pneumococcal vaccination was not found to be an increased risk, contrary to prior suggestions [57, 58]. Arias et al. suggested that antigens injected into or near the synovial joint may lead to a localized inflammatory response in rare cases [58]. Other studies have reported that maladministered influenza vaccine is a risk factor for rotator cuff tears [52, 53]. Hessee et al. recommend training in proper vaccination administration techniques to ensure the injection enters the deltoid muscle and avoids the bursa, tendons, ligaments, and bones of the upper shoulder. As this association was observed for rotator cuff tears, future investigations should include a non-shoulder joint disorder as a comparison group. The general anti-viral class of medications and barbiturate medications were not found associated with AC in this analysis. Barbiturate medications were generally not covered by Medicare in 2011 [59], which may explain the small numbers of cases in the data. The other classes of investigated medications (retinoids, protease inhibitors, metalloproteinases, isoniazid) were prescribed to 0.5% or fewer of Medicare recipients at large [60] and were too infrequent in this dataset to assess as risk factors.

No positive association was found between AC diagnosis and five classes of infections in the six months prior to diagnosis. Those with an upper respiratory tract infection were less likely to be diagnosed with AC. Similarly, sustaining a non-shoulder related fracture or sprain or another injury was not associated with AC. The claims data do not allow in depth analysis, but it is possible rotator cuff tears could be due to trauma rather than these factors being protective against AC.

AC was more commonly diagnosed August through December. However, no significant seasonal trend was observed for AC, contrary to earlier suggestions [33, 61]. No medical records were available to determine whether the Medicare claim date for first AC visit reflects the date the condition began or the date when care was sought.

## Conclusions

While this investigation did not identify specific triggers for AC, it did corroborate the long-known associations with diabetes and Parkinson’s disease in the Medicare population. Future investigations should investigate initiating triggers for the development of this common and disabling condition.

## Data Availability

The data that support the findings of this study are available in their original form from ResDAC but restrictions apply to the availability of these data, which were used under license for the current study, and so are not publicly available. Data are available from the authors upon reasonable request and with permission from the Center for Effectiveness Research in Orthopaedics and the University of South Carolina.

## Abbreviations

AC: Adhesive capsulitis
ATC4: Anatomical Therapeutic Chemical -4 Classification
CPT®: Current Procedural Terminology
FFS: Fee-for-service medical care
HCPCS: Healthcare Common Procedure Coding System
ICD-9-CM: International Classification of Disease, Ninth Revision, Clinical Modification
MRI: Magnetic resonance imaging
RCT: Rotator cuff tear
